# Case Report: Thymidine Kinase 2 (TK2) Deficiency: A Novel Mutation Associated With Childhood-Onset Mitochondrial Myopathy and Atypical Progression

**DOI:** 10.3389/fneur.2022.857279

**Published:** 2022-02-25

**Authors:** Arianna Manini, Megi Meneri, Carmelo Rodolico, Stefania Corti, Antonio Toscano, Giacomo Pietro Comi, Olimpia Musumeci, Dario Ronchi

**Affiliations:** ^1^Dino Ferrari Center, Neuroscience Section, Department of Pathophysiology and Transplantation, University of Milan, Milan, Italy; ^2^Neurology Unit, IRCCS Foundation Ca' Granda Ospedale Maggiore Policlinico, Milan, Italy; ^3^Unit of Neurology and Neuromuscular Disorders, Department of Clinical and Experimental Medicine, University of Messina, Messina, Italy; ^4^Neuromuscular and Rare Diseases Unit, Department of Neuroscience, Fondazione IRCCS Ca' Granda Ospedale Maggiore Policlinico, Milan, Italy

**Keywords:** thymidine kinase 2, TK2, mitochondrial DNA, mtDNA maintenance defects, myopathy, deoxynucleosides

## Abstract

The nuclear gene *TK2* encodes the mitochondrial thymidine kinase, an enzyme involved in the phosphorylation of deoxycytidine and deoxythymidine nucleosides. Biallelic *TK2* mutations are associated with a spectrum of clinical presentations mainly affecting skeletal muscle and featuring muscle mitochondrial DNA (mtDNA) instability. Current classification includes infantile- ( ≤ 1 year), childhood- (1–12 years), and late-onset (≥12 years) forms. In addition to age at onset, these forms differ for progression, life expectancy, and signs of mtDNA instability (mtDNA depletion vs. accumulation of multiple mtDNA deletions). Childhood-onset TK2 deficiency typically causes a rapidly progressive proximal myopathy, which leads to wheelchair-bound status within 10 years of disease onset, and severe respiratory impairment. Muscle biopsy usually reveals a combination of mitochondrial myopathy and dystrophic features with reduced mtDNA content. Here we report the case of an Italian patient presenting childhood-onset, slowly progressive mitochondrial myopathy, ptosis, hypoacusis, dysphonia, and dysphagia, harboring the *TK2* variants c.278A>G and c.543del, the latter unreported so far. Compared to other childhood-onset *TK2*-patients, our case displays atypical features, including slowly progressive muscle weakness and absence of respiratory failure, which are usually observed in late-onset forms. This report extends the genetic background of TK2-related myopathy, highlighting the clinical overlap among different forms.

## Introduction

Mitochondrial DNA (mtDNA) maintenance defects are a heterogeneous group of clinical syndromes characterized by mtDNA deletions and/or depletion and derived from mutations in nuclear genes variably involved in mtDNA homeostasis (i.e., *POLG1, POLG2, TWNK, DGUOK, TYMP*) (1–5).

In 2001, Saada et al. (6) detected biallelic mutations in the thymidine kinase 2 (*TK2*) gene in four children presenting severe myopathy associated with muscle mtDNA depletion. TK2 is a nuclear-encoded mitochondrial enzyme involved in the phosphorylation of deoxycytidine and deoxythymidine nucleosides, which represents the first step of a salvage pathway aimed to provide deoxyribonucleotides (dNTPS) for mtDNA synthesis (7). Recessive *TK2* mutations are now an established cause of mtDNA maintenance disorders, recently classified on the basis of clinical and biochemical features (8). According to age at onset, TK2 deficiency can lead either to an infantile-onset ( ≤ 1 year), childhood-onset (1–12 years) and late-onset (≥12 years) myopathy, which differ for rate of weakness progression, post-onset survival and predominance of mtDNA multiple deletions or depletion in muscle tissue, as summarized in Table 1 (8). Childhood-onset TK2 deficiency is typically associated with rapidly progressive, proximal myopathy, which leads to wheelchair-bound status within 10 years of disease onset in most patients, and post-onset survival longer than 13 years (8). More than half of childhood-onset TK2 patients require ventilatory support due to respiratory impairment (8). Ptosis, chronic external ophthalmoplegia (CPEO), facial weakness and dysphagia have been reported less frequently in this group of patients compared to late-onset cases (8–11), while cognitive decline, encephalopathy, seizures, and non-muscle manifestations are rare compared to the infantile-onset form (8–10). Needle electromyography (EMG) examination frequently evidences myopathic changes [i.e., polyphasic, short-duration, low-amplitude motor unit potentials (MUPs)] (8). Muscle histology reveals a combination of mitochondrial dysfunction [i.e., cytochrome C oxidase (COX)-negative fibers; ragged-red fibers (RRF)] and, mainly in pediatric cases, dystrophic features (i.e., atrophic fibers, fibrosis and increase of connective tissue) (8, 12, 13). Infantile and childhood-onset cases often showed mtDNA depletion in muscle tissue, whereas the late-onset ones are usually associated with mtDNA multiple deletions (8).

**Table 1:** Clinical and biochemical features associated with the three different phenotypes of TK2-related mitochondrial myopathy and mtDNA maintenance defects [data were obtained from Garone et al. (8)].

	**Infantile-onset**	**Childhood-onset**	**Late-onset**
**Age at onset**	≤ 1 year	1-12 years	≥12 years
**Prevalence among TK2-related mitochondrial myopathy cases**	43%	41%	16%
**Myopathy**	Severe, congenital, rapidly progressive	Moderately to rapidly progressive	Subtle signs of myopathy in childhood; slowly progressive; like facioscapulohumeral dystrophy
**Ptosis and PEO**	8%	30%	69%
**Progression to wheelchair-bound status**	4 years or no ability to walk (94%)	10 years (63%)	No
**Respiratory impairment**	+++	++	+
**Ventilatory support**	89%	55%	44%
**Nervous system involvement**	26%	11%	0%
**Additional neurological features**	Seizures (18%) Encephalopathy (13%) Cognitive dysfunction (8%) Facial diplegia (8%) Dysphagia (8%) Lissencephaly (3%) Microcephaly (3%) Bilateral optic atrophy (3%)	Facial diplegia (30%) Hypoacusis (5%) Dysphagia (3%) Cognitive decline (3%) Encephalopathy (3%)	Facial diplegia (43%) Dysphagia (43%) Dysarthria/dysphonia (21%) Peripheral neuropathy (7%) Hypoacusis (7%)
**Non-skeletal muscle involvement**	33%	19%	25%
**Additional non-neurological features (rare)**	Multiple bone fractures (5%) Nephropathy (3%) Rigid spine (3%) Cardiomyopathy (3%) Bi-ventricular hypertrophy (3%) Arrhythmia (3%) Esophageal atresia (3%) Anemia (3%) Capillary-leak syndrome (3%) Bilateral chylothorax (3%) Occipital skin necrosis (3%)	Prolonged QT (3%) Arrhythmia (3%) Multiple bone fractures (3%) Renal tubulopathy (3%) Gynecomastia (3%)	Cardiomyopathy (14%)
**mtDNA depletion**	81%	77%	7%
**mtDNA multiple deletions**	12.5%	50%	100%
**Post-onset survival**	1 year	23 years (compound with late-onset cases)	23 years (compound with childhood-onset cases)

*MtDNA, mitochondrial DNA; PEO, progressive externa ophthalmoplegia*.

Herein, we report the case of an Italian patient affected by a childhood-onset, slowly progressive mitochondrial myopathy with atypical clinical features, harboring two heterozygous *TK2* variants, one of which has not been reported before.

## Case Description

The proband is a 55-year-old Italian woman from Sicily, born to non-consanguineous parents. Mild bilateral ptosis and dysphonia were noticed starting from 8 years of age. At 20 years of age, she started to complain of dysphagia of both solid food and liquids and began losing weight. Over the years she developed bilateral upper limb weakness and mild hypoacusis, with slow progression in the last 20 years. Cognitive abilities are normal.

Current clinical examination reveals bilateral ptosis and ophthalmoparesis, myopathic face, marked dysphonia, wasting, weakness of proximal upper limbs and of psoas muscles, more prominent on the right.

**Figure 1:**
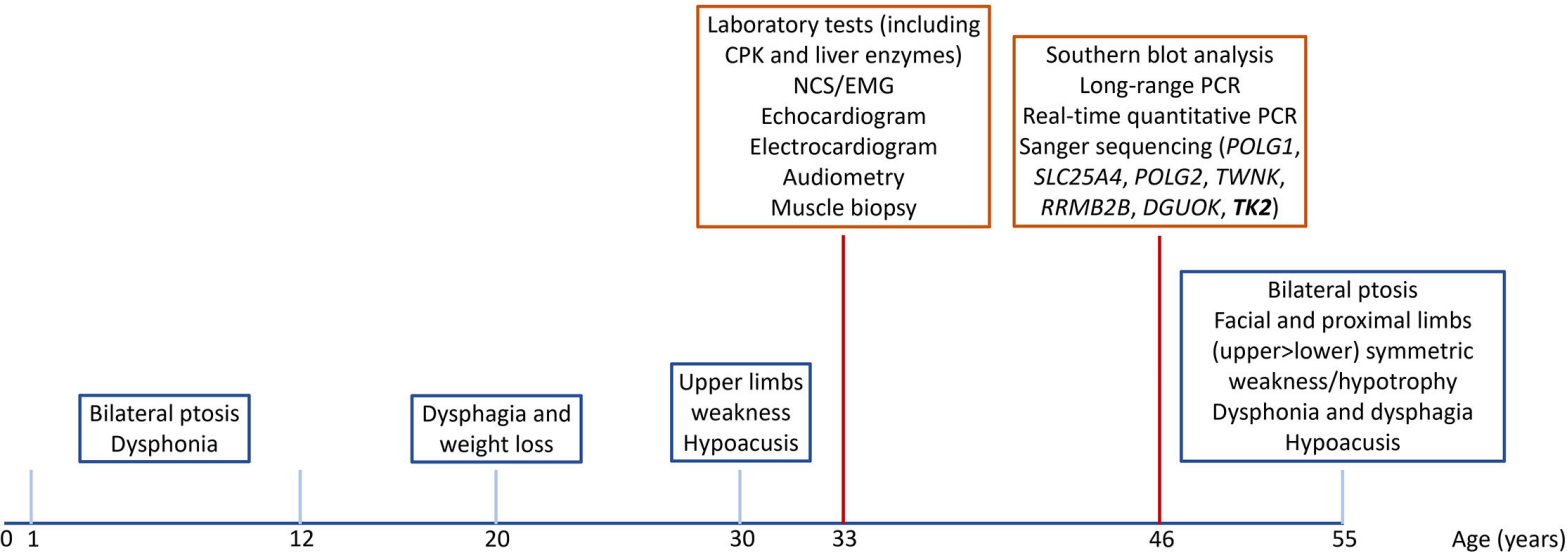
Timeline of relevant clinical signs and symptoms (blue squares), and of diagnostic assessments (red squares).

The complete timeline of relevant clinical signs and symptoms and of diagnostic assessments performed during disease progression is reported in Figure 1.

Creatine phosphokinase (CK) levels are currently 251 IU/L (normal value <200). During first hospitalization at 32 years of age, CK levels reached 3,880 IU/L, with concomitant increase of liver enzymes levels, including aspartate aminotransferase (AST) (243 IU/L; normal value <43 IU/L), alanine aminotransferase (ALT) (98 IU/L; normal value <45 IU/L) and lactate dehydrogenase (LDH) (1,459 IU/L; normal value <300 IU/L). Lactate levels were only mildly increased at baseline (2.6 mmol/L; normal value <1.5 mmol/L), but significantly raised under workload (4.6 mmol/L; normal value <2.3 mmol/L).

Needle EMG examination, performed at 33 years, showed rapid recruitment of short-duration, low-amplitude MUPs in bilateral biceps and first interosseous muscles. Audiometry, electrocardiogram, echocardiogram and spirometry were normal. At the same age, she underwent muscle biopsy of left biceps, which revealed increased fibers size, with coexistence of both atrophic and hypertrophic fibers in addition to mitochondrial dysfunction features, including 10% RRF, succinate dehydrogenase (SDH) hyperactive fibers and COX-negative fibers. The adenosine triphosphatase (ATPase) staining showed marked prevalence of type 1 fibers (almost 90%), and, to a lesser extent, type 2c fibers.

**Figure 2:**
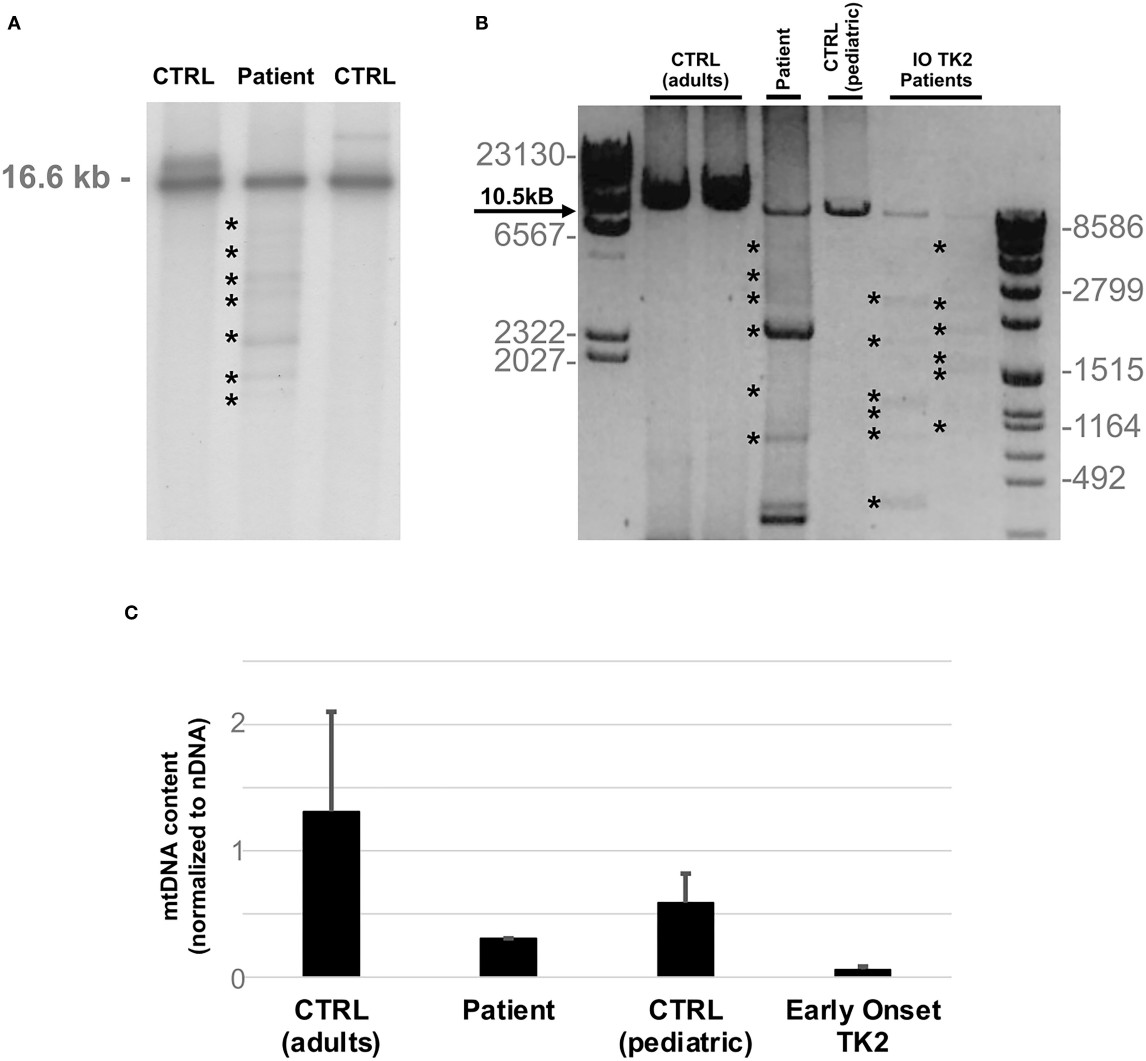
MtDNA studies. **(A)** Southern blot analysis of mtDNA obtained from patient's muscle biopsy and age matched controls. Asterisks indicate multiple bands corresponding to partially deleted mitochondrial genomes. The expected size of normal linearized mtDNA is indicated. **(B)** Long-range PCR analysis of mtDNA obtained from patient's muscle biopsy and two previously described infantile onset TK2 patients (IO TK2) compared to respective age matched controls. Asterisks indicate multiple bands corresponding to multiple mtDNA deletions. Black arrow indicates the expected size of a wild-type PCR amplicon (10.5 kB: FOR5635-RC16135). The sizes of the bands of the ladders (DNA Molecular Weight Marker II and VII, Roche) are indicated. **(C)** Histogram plot showing muscle mtDNA content in patient's muscle and two previously described infantile onset TK2 patients compared to respective age matched controls (*n* = 8, each group). Bars represent mean values. Error bars indicate standard deviation. MtDNA quantification, normalized to nuclear DNA (nDNA) content, was performed by quantitative PCR.

Southern blot analysis and long-range polymerase chain reaction (PCR) of muscle mtDNA showed multiple mtDNA deletions (Figures 2A,B). Quantitative PCR showed loss of mtDNA integrity (30% compared to age-matched controls; Figure 2C).

After excluding mutations in the common genes associated with multiple mtDNA deletions (*POLG1, SLC25A4, POLG2, TWNK, RRMB2B, DGUOK*), direct sequencing of *TK2* (NM_004614.4) revealed the heterozygous mutations c.278A>G and c.543del resulting in protein changes p.Asn93Ser and p.Leu182Phefs^*^11, respectively. The c.278A>G variant [rs142291440; Genome Aggregation Database (gnomAD) Minor Allele Frequency (MAF) 1.2 × 10^−6^] has been previously detected in an African American patient affected by childhood-onset mitochondrial myopathy (14). The microdeletion c.543del is absent in publicly available databases. DNA from relatives was not available for molecular studies.

## Discussion

We herein describe the case of an Italian patient from Sicily affected with a mild form of childhood-onset mitochondrial myopathy, with muscle mtDNA multiple deletions and depletion, associated with two heterozygous mutations in *TK2*, one of which is novel. To date, this is the second Italian case of *TK2*-related myopathy described so far (15). Intriguingly, both families are of Sicilian origin.

Both the p.Asn93Ser and p.Leu182Phefs^*^11 variants are located within the deoxynucleoside kinase domain, which spans amino acids 53–260. This large functional domain is involved in the phosphorylation of deoxypyrimidine nucleosides, the first step of the mtDNA synthesis cascade (7). The p.Asn93Ser has been already reported by Oskoui et al. (14). Both our proband and the patient described by Oskoui and colleagues display childhood-onset myopathy with progressive development of fatigue, proximal muscle weakness, and facial diplegia, and similar levels of mtDNA depletion assessed in muscle tissue. Main differences between the two cases are represented by the absence of ptosis and the presence of neuropathic changes at EMG reported by Oskoui and colleagues (14). The novel p.Leu182Phefs^*^11 variant is located in exon 8, which encodes the α8 helix involved in the catalysis of adenosine triphosphate (ATP) molecules due to its capacity of binding phosphate groups. Exon 8 is the second hot spot of *TK2* pathogenic variants, following exon 5 (8). Small indels causing frameshift represent the second most prevalent type of *TK2* variants in mtDNA maintenance defects (13%), following missense mutations (66%), and are distributed throughout *TK2* length (16).

Consistently with classical cases of childhood-onset *TK2* deficiency, analysis of mtDNA levels in muscle tissue of our patient demonstrated both depletion and multiple deletions, and CK levels were mildly elevated (8). Muscle histology findings included non-specific myopathic changes (i.e., fiber size variability, with type 1 predominance), and mitochondrial myopathy markers without dystrophic features. However, several clinical features are atypical compared to previous reports of childhood-onset *TK2*-related myopathy (Table 1). First, muscle weakness has remained stable over the last 20 years, and our patient is currently able to walk without support. Childhood-onset *TK2*-related myopathy is usually rapidly progressive, and patients become wheelchair-bound within 10 years of disease onset. In the retrospective analysis of natural history data of genetically confirmed TK2 deficient patients performed by Garone and colleagues, only 4 out of 30 childhood-onset patients were able to walk independently after 10 years from disease onset (8). Of the remaining childhood-onset cases, 19 (63%) became wheelchair-bound within 10 years of disease onset, and 8 (27%) were within 10 years of disease onset, so that their muscle impairment rate of progression could not be assessed. Second, respiratory failure with invasive or non-invasive ventilatory dependency is frequent, reaching about half childhood-onset cases (8). At 55 years of age, our patient has not developed respiratory impairment. Third, dysphagia, which has been described in our case, is rare in childhood-onset *TK2*-related myopathy, especially compared to late-onset forms (8).

As deoxynucleosides therapies for TK2 deficiency are under investigation, identifying patients carrying *TK2* mutations affected by mtDNA maintenance defects becomes pivotal (17). Domínguez-Gonzàlez and colleagues have recently reported the results of an open-label study in which pyrimidine deoxynucleosides and deoxynucleotides were orally administered to 16 patients with mitochondrial myopathy due to TK2 deficiency (17). In early-onset patients affected with severe forms of myopathy, the treatment was effective in increasing survival and ameliorating muscle weakness, respiratory function, and dysphagia, with no major side effects (17). On the contrary, the beneficial effects of the administration of pyrimidine deoxynucleosides and deoxynucleotides in adult-onset cases were limited, and hepatic toxicity was suspected in two patients (17).

The application of NGS-based sequencing in a clinical setting is rapidly expanding the number of novel diagnoses in mitochondrial disorders. On the other hand, the identification of specific changes (such as the coexistence of mtDNA depletion and deletions) might restrict the number of genes to be investigated to those involved in mitochondrial dNTPs supply pathways. Timely or early molecular diagnosis for TK2 patients is crucial for the recruitment in the ongoing clinical trials and the access to rescue therapies in the near future.

## Data Availability Statement

The raw data supporting the conclusions of this article will be made available by the authors, without undue reservation.

## Ethics Statement

The studies involving human participants were reviewed and approved by the Comitato Etico Milano Area 2 Fondazione IRCCS Ca' Granda Ospedale Maggiore Policlinico (Milan, Italy). The patients/participants provided their written informed consent to participate in this study. Written informed consent was obtained from the individual(s) for the publication of any potentially identifiable images or data included in this article.

## Author Contributions

AM and DR interpreted the results, conceived the idea, revised the literature, and wrote the manuscript. DR performed genetic analysis and mtDNA studies. OM made the clinical evaluation. OM, MM, CR, AT, GC, and SC performed a critical revision of the manuscript for important intellectual content. All the authors have read and approved the manuscript.

## Funding

This study was funded by Italian Ministry Fundation IRCCS Ca' Granda Ospedale Maggiore Policlinico Ricerca Corrente 2020 to GC. This work was promoted within the European Reference Network (ERN) for Neuromuscular Diseases.

## Conflict of Interest

The authors declare that the research was conducted in the absence of any commercial or financial relationships that could be construed as a potential conflict of interest.

## Publisher's Note

All claims expressed in this article are solely those of the authors and do not necessarily represent those of their affiliated organizations, or those of the publisher, the editors and the reviewers. Any product that may be evaluated in this article, or claim that may be made by its manufacturer, is not guaranteed or endorsed by the publisher.
